# Relapsing-Remitting Multiple Sclerosis Is Associated With Regional Brain Activity Deficits in Motor- and Cognitive-Related Brain Areas

**DOI:** 10.3389/fneur.2019.01136

**Published:** 2019-11-26

**Authors:** Xiao-Feng Du, Jiao Liu, Qi-Feng Hua, Yi-Jiao Wu

**Affiliations:** Department of Radiology, Hwa Mei Hospital, University of Chinese Academy of Sciences, Ningbo, China

**Keywords:** cognitive function, fractional amplitude of low-frequency fluctuation, functional magnetic resonance imaging, motor system, multiple sclerosis, receiver operating characteristic

## Abstract

**Objective:** To identify the abnormal regional spontaneous brain activity associated with relapsing-remitting multiple sclerosis (RRMS) using fractional amplitude of low-frequency fluctuation (fALFF) analysis and their relationships with clinical features.

**Methods:** A total of 26 RRMS (11 males, 15 females; age, 36.58 ± 10.82 years) and 27 status-matched healthy group (HGs; 12 males, 15 females; age, 35.85 ± 12.05 years) underwent an Expanded Disability Status Scale (EDSS) examination. fALFF was applied to evaluate the abnormal regional brain activity associated with RRMS. Pearson's correlation analysis was applied to calculate the correlations between the signal values of brain areas that exhibited abnormal fALFF values and clinical features. Receiver operating characteristic (ROC) curve was performed to evaluate the sensitivity and specificity of those altered brain areas to distinguish between RRMS and HGs.

**Results:** Compared with HGs, RRMS exhibited higher fALFF in the right cerebellum posterior lobe, left orbitofrontal cortex, left dorsolateral prefrontal cortex, bilateral supplementary motor area, and right fusiform gyrus and lower fALFF values in the left hippocampus and right precuneus. ROC revealed that these areas showed two good and five fair AUC values (0.77 ± 0.03, 0.729~0.822). However, four combinations with more than five brain regions received the same best discriminatory power with a sensitivity of 96.3% and a specificity of 88.5%. EDSS revealed a negative correlation with supplementary motor area (*r* = −0.395, *p* = 0.046).

**Conclusions:** RRMS is associated with abnormal regional brain activity deficits of motor- and cognitive-related areas. The fALFF parameter may serve as a potential biological marker to discriminate between the two groups.

## Introduction

Multiple sclerosis (MS) is a demyelinating disease in axonal degeneration with commonly diagnosed in the prime of life. In addition to classic inflammatory white matter lesions, extensive gray matter demyelination have been gradually known (1, 2). Extensive involvements of clinical symptoms-related (3, 4) gray matter abnormalities could be found by routine and functional magnetic resonance imaging (MRI) (5–7), these disease-related cortical abnormalities may delay and perturb syntonic intrinsic signals across cortico-cortical and cortico-subcortical wirings and lead to several clinical manifestations, such as fatigue, sensorimotor deficits, cognitive dysfunction, even chronic disability (2). Although extensive neurotoxic effects of MS on brain and behavior have been known, the neurobiological mechanism of complex brain network impairments underlying MS remains ambiguous.

It is proposed that resting-state functional MRI (rs-fMRI) is a useful tool for the mechanism research of brain disease (8) and exhibited an effective way to characterize the resting-state brain activity without any tasks that may represent more than 95% of the total activity of the brain (9). The rs-fMRI can be used to describe the regional spontaneous neuronal brain activity without exposing to the radioactive tracers (8). In contrast to magnetoencephalography and conventional electrophysiologic methods, the rs-fMRI has the capacity to map the physiological effects of neuronal activity with high spatial resolution. Cordani et al. found widespread gray matter atrophy, microstructural white matter abnormalities, and decreased resting state functional connectivity in motor and cognitive networks in patients with MS, which contribute to explain motor disability in MS patients (10). Plata-Bello et al. found a decrease of amplitude of low-frequency fluctuations (ALFF) in the left inferior frontal gyrus in MS patients, and this area correlated with the gray matter volume of the left inferior parietal lobule (11). van Geest et al. found that compared to non-depressed MS patients, depressed MS patients had lower white matter volume (*p* < 0.01), decreased fractional anisotropy of the uncinate fasciculus (*p* < 0.05), and lower resting-state functional connectivity between the amygdala and the frontal regions (*p* < 0.05) (12). Furthermore, the disease duration, fractional anisotropy of the uncinate fasciculus, and the functional connectivity pair could explain 48% of variance in the severity of depression. These findings help us understand the pathophysiology of the MS.

The ALFF and fractional ALFF (fALFF) are two important parts of the rs-fMRI. The spontaneous LFFs have been suggested to arise from neurovascular mechanisms (13). Although the nature of the LFFs remains unclear, the proposed ALFF method (14) may be associated with regional spontaneous neuronal activity (15) and has served as a sensitive biological indicator to discriminate one group from the other group (16). However, this method is sensitive to physiological noise, which may hamper us to describe the alterations of regional spontaneous neuronal brain activity. The fALFF may overcome this limitation (17). The improved method could provide a more specific index of low-frequency oscillatory phenomena by measuring local fluctuations in neuronal activity rather than generalized neuronal activity (18). The proposed new method of fALFF has been shown to decrease bias and increase sensitivity relative to the ALFF method (17). Recently the fALFF method has been widely used to explore the neurobiological mechanism for several diseases, such as schizophrenia (19), depressive disorder (20), and Alzheimer's disease (21). Liu et al. (22) found that abnormal regional brain activity was detected in patients with MS with simple spinal cord involvement by this method. However, relapsing-remitting MS (RRMS) has not been studied by this method. RRMS is associated with changes in behavior, brain function, and brain structure. However, the nature of these changes have not been well-understood. Motivated by previous work, in the present study, it is the first to utilize the fALFF method to identify the regional spontaneous brain activity associated with RRMS.

## Materials and Methods

### Subjects

Total of 26 RRMS (11 males, 15 females; mean age, 36.58 ± 10.82 years; mean education, 10.69 ± 2.53 years) and 27 age-, sex-, and education-matched healthy groups (HGs; 12 males, 15 females; mean age, 35.85 ± 12.05 years; mean education, 11.07 ± 2.85 years) were recruited.

The RRMS met the pertinent inclusion criteria: (1) met the diagnostic criteria of RRMS as defined by McDonald; (2) had mild to moderate RRMS as defined by the Expanded Disability Status Scale (EDSS) examination (EDSS ≤ 5), to prevent confounding factors in the analysis; (3) had not received steroids-based therapy or experiencing a clinical relapse, or other concomitant therapy as antidepressant or therapy for fatigue for at least 2 months prior and during this study; (4) had no sleep disorders and other psychiatric disorders, as defined by the DSM-IV; (5) had no brain tumor, brain injury, intracerebral hemorrhage, according to the conventional MRI. The disease duration was recorded in months from the symptom onset date to the MRI scan date.

The HGs had no history of brain tumor, brain injury, inborn or other acquired diseases, neurological or psychiatric disorders, sleep disorders, alcohol abuse, drug abuse, systemic illness, intracerebral hemorrhage, and foreign implants in the body. All subjects were right-handed, as defined by the Edinburgh Inventory.

All volunteers underwent a thorough physical examination using the EDSS by an experienced neurologist. The EDSS score is from 0 to 10, where zero score indicates normal, and higher EDSS score shows more disability. All volunteers participated voluntarily and were explained the purposes, methods, and potential risks. Before the MRI session, all subjects voluntarily signed their informed consent form. This study was approved by the Human Research Ethics Committee.

### MRI

MRI scan was performed on 3-Tesla MR scanners (Trio, Siemens, Erlangen, Germany). High-resolution T1-weighted anatomical images were acquired with a three-dimensional magnetization prepared rapid-gradient-echo sequence in a sagittal orientation: the brain volume with 176 slice phase encoding directions (repetition time = 2,300 ms, echo time = 2.98 ms, slice thickness = 1.0 mm, acquisition matrix = 256 × 256, field of view = 256 mm × 256 mm, flip angle = 9°) were obtained. Total of 240 functional images (repetition time = 2,000 ms, echo time = 30 ms, slice thickness = 4.0 mm, gap = 1.2 mm, acquisition matrix = 64 × 64, flip angle = 90°, field of view = 220 mm × 220 mm, 32 slices) covering the whole brain were obtained, which took about 8 min for functional data acquisition. Other sequences, such as T1WI, T2WI, T2 FLAIR, and diffusion-weighted imaging, were also acquired.

### Data Analysis

The first 10 time points of the functional images were discarded due to the possible instability of the initial MRI signal and inadaptation to the scanning environment (23). On the basis of MATLAB2010a (Mathworks, Natick, MA, USA), data pre-processing of the remaining resting-state images was performed by Data Processing & Analysis for Brain Imaging (DPABI 2.1, http://rfmri.org/DPABI) toolbox (24), including form transformation, slice timing, head motion correction, spatial normalization, and smooth with a Gaussian kernel of 6 × 6 × 6 mm^3^ full-width at half-maximum. The 3D T1-weighted images for each subject were coregistered to the mean of the realigned EPI template and then segmented into gray matter, white matter, and cerebrospinal fluid (CSF) using the Diffeomorphic Anatomical Registration Through Exponentiated Lie Algebra (DARTEL) segmentation. Participants with more than 1.5 mm maximum translation in x, y, or z directions and 1.5° degree of motion rotation were removed. The Friston 24 head motion parameters model, including six head motion parameters, six head motion parameters, and 12 corresponding squared items, was used to regress out the head motion effects based on recent work showing that the higher-order models benefit from the removal of head motion effects (25–28). In RRMS, T1-hypointense lesions were manually identified and were semiautomated painted as regions of interest (ROIs) using the T1 sagittal images converted to axial. Lesion masks for each patient were created (transforming the ROIs into independent images) and then binarized using ImCalc module. After this step, the binary lesion masks together with the T1 sagittal images were used to create new structural images. The created images were automatically transformed from the individual space to the Montreal Neurological Institute (MNI) space. Linear regression was applied to remove other sources of spurious covariates along with their temporal derivatives, including the global mean signal, white matter and cerebrospinal fluid signal. After the head-motion correction, the functional MRI images were spatially normalized to the MNI space and resampled at a resolution of 3 × 3 × 3 mm^3^. After pre-processing, the time series for each voxel were linearly detrended to reduce the low-frequency drift and physiological high-frequency respiratory and cardiac noise.

The ALFF measure at each voxel was the averaged square root of the power spectrum of low-frequency (0.01–0.08 Hz). The fALFF value was the ratio of this power spectrum to entire frequency range. The fALFF was calculated by DPABI toolbox.

### Statistical Analysis

The demographic factors (age, education, and years of education) and the questionnaire data were compared between groups using two sample *t*-test. Chi-square (χ^2^) test was used for categorical data. The statistical analysis was performed using IBM Statistical Package for the Social Sciences version 21.0 (SPSS 21.0). Data are presented as mean ± standard deviation. All results were quoted as two-tailed, and *P* < 0.05 was considered as statistically significant.

Two-sample *t*-tests were used to investigate the fALFF differences in brain regions between RRMS and HGs with age, sex, and years of education as nuisance covariates of no interest. A corrected threshold of two-tailed voxel-wise *p* < 0.001 and cluster-level *p* < 0.001, corrected for multiple comparisons by false discovery rate (FDR), was used for one-sample *t-*tests. A loose corrected threshold of two-tailed voxel-wise *p* < 0.01 and cluster-level *p* < 0.05 with a minimum continuous cluster voxel volume of 540 mm^3^ was used for two-sample *t-*tests.

Recently, ROC curve was increasingly applied into the exploration of the reliability of one neuroimaging parameter as a potential biological indicator in distinguishing one group from another group (8, 16, 29–31). The mean beta values of those brain regions exhibiting abnormal fALFF in RRMS relative to HGs were entered into ROC curve analysis. Pearson correlation analysis was performed to evaluate the relationships between the behavioral performances (disease duration and EDSS score) and the fALFF value of those different brain regions. *p* < 0.05 was considered to be significant differences.

## Results

### Sample Characteristics

The demographic characteristics of the subjects are presented in Table 1. There were no significant differences in sex (χ^2^ = 0.025, *p* = 0.875), mean age (*t* = 0.23, *p* = 0.819), and mean education (*t* = −0.515, *p* = 0.609) between RRMS and HGs. The mean disease duration and EDSS score of RRMS was 22.02 (±32.96) days and 3.62 (±1.8), respectively.

**Table 1:** Demographics and clinical characteristics.

	**RRMS**	**HGs**	**t/χ^2^ value**	***p*-value**
Sex (Male, Female)	11/15	12/15	0.025	0.875^*^
Age, year	36.58 ± 10.82	35.85 ± 12.05	0.23	0.819 ^#^
Education, year	10.69 ± 2.53	11.07 ± 2.85	−0.515	0.609 ^#^
Disease duration, day	22.02 ± 32.96	N/A	N/A	N/A
EDSS score	3.62 ± 1.8	N/A	N/A	N/A

Data are mean ± standard deviation values;

* χ^2^ test was used;

# *independent t-test. RRMS, relapsing-remitting multiple sclerosis; HGs, healthy groups; N/A, not applicable; EDSS, Expanded Disability Status Scale*.

### fALFF Differences Between Groups

**Figure 1:**
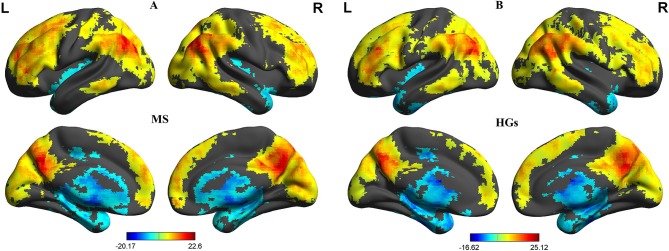
One sample *t*-tests of fALFF differences. One sample *t*-tests of fALFF differences in MS **(A)** and HGs **(B)**. RRMS, relapsing-remitting multiple sclerosis; HGs, healthy groups; R, right; L, left; fALFF, fractional amplitude of low-frequency fluctuation.

**Figure 2:**
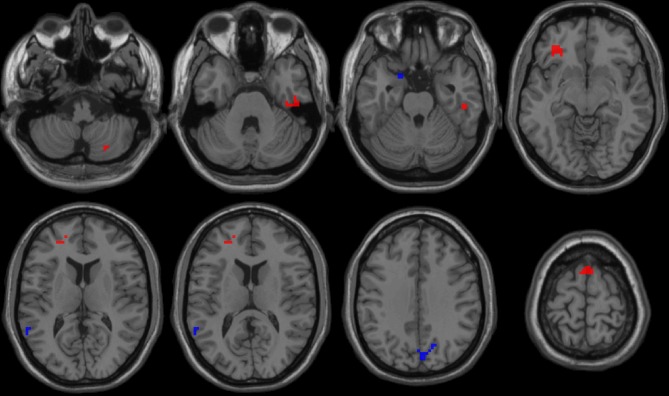
fALFF differences between RRMS and HGs. RRMS, relapsing-remitting multiple sclerosis; HGs, healthy groups; R, right; L, left; fALFF, fractional amplitude of low-frequency fluctuation.

First, we reported within-group statistic maps for fALFF measurement for RRMS (Figure 1A) and HGs (Figure 1B) using one sample *t*-test (*p* < 0.001, FDR corrected). We found that the two groups showed significantly similar fALFF value in brain areas, but the covered areas in RRMS are wider than those in HGs (Figure 2). Next, the fALFF differences between RRMS and HGs were compared. Compared with HGs, RRMS exhibited significantly higher fALFF values in the right cerebellum posterior lobe, left orbitofrontal cortex (BA 47), left dorsolateral prefrontal cortex (BA 46) in the salience network, bilateral supplementary motor area (BA 6) in the salience network, and right fusiform gyrus (BA20) and lower fALFF values in the left hippocampus (BA28) and right precuneus (BA 7) in the occipital cortex (Figure 2; Table 2).

**Table 2:** fALFF differences between RRMS and HGs.

**Conditions**	**Brain regions of peak coordinates**	**R/L**	**BA**	**Voxel volume (mm^**3**^)**	***t*-score of peak voxel**	**MNI coordinates**		
						**X**	**Y**	**Z**
RRMS > HGs	Cerebellum posterior lobe	R	N/A	594	3.5264	24	−72	−51
RRMS > HGs	Inferior frontal gyrus	L	47	1,026	4.3785	−33	36	−9
RRMS > HGs	Superior frontal gyrus	L	46	756	3.583	−21	48	24
RRMS > HGs	Superior frontal gyrus	L, R	6	540	3.5727	6	15	69
RRMS > HGs	Fusiform gyrus	R	20	810	3.4781	42	−24	−30
RRMS < HGs	Precuneus	R	7	648	−3.5184	15	−66	33
RRMS < HGs	Hippocampus	L	28	540	−8.3636	−21	12	−33

*The statistical threshold was set at uncorrected voxel threshold of p < 0.01 with a minimum voxel volume threshold of 540 mm^3^. fALFF, fractional amplitude of low-frequency fluctuation; RRMS, relapsing-remitting multiple sclerosis; HGs, healthy groups; R, right; L, left; BA, Brodmann's area; MNI, Montreal Neurological Institute; N/A, not applicable*.

### Pearson Linear Correlation

**Figure 3:**
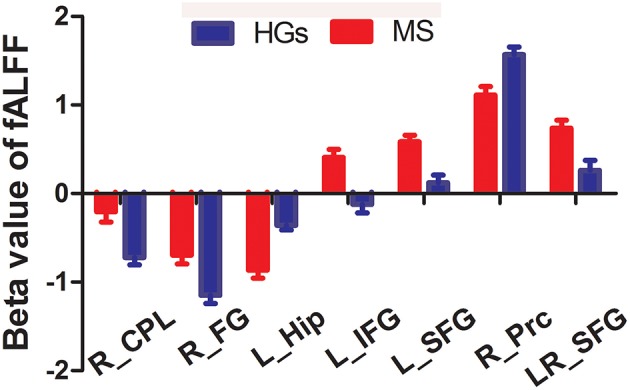
Beta value of between-group differences in fALFF. Data are mean ± standard error values. fALFF, fractional amplitude of low-frequency fluctuation; R, right; L, left; MS, multiple sclerosis; HGs, healthy groups; CPL, cerebellum posterior lobe; FG, fusiform gyrus; Hip, hippocampus; IFG, inferior frontal gyrus; SFG, superior frontal gyrus; Prc, precuneus.

**Figure 4:**
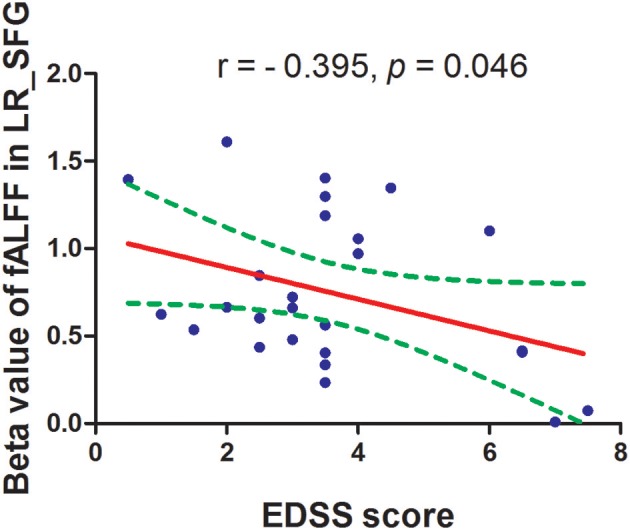
Pearson's correlation between EDSS score and beta value of caudate head. fALFF, fractional amplitude of low-frequency fluctuation; EDSS, Expanded Disability Status Scale.

The mean fALFF values of these different areas were extracted (Figure 3) and used for Pearson linear correlation analysis. As shown in Figure 4, EDSS revealed a negative linear correlation with fALFF value in the bilateral supplementary motor area (BA 6) in the salience network (*r* = −0.395, *p* = 0.046), but no significant difference can be found after multiple testing. No other significant linear correlations between fALFF value in those different brain areas and the behavioral performances were found (*p* > 0.05).

### High Sensitivity and Specificity

**Figure 5:**
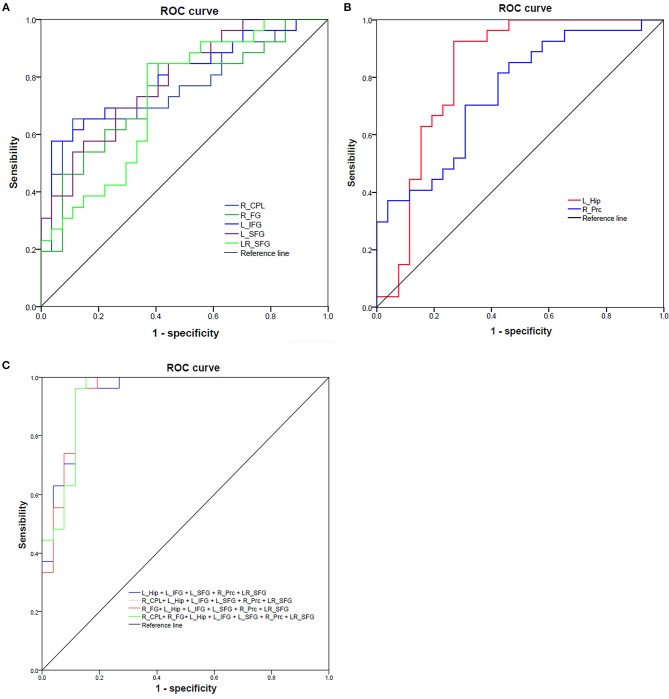
ROC curve of mean fALFF values value of those different brain regions between RRMS and HGs. ROC curve of increased **(A)** and decreased **(B)** fALFF differences and four combinations with more than five brain regions **(C)**. ROC, receiver operating characteristic; R, right; L, left; RRMS, relapsing-remitting multiple sclerosis; HGs, healthy groups; CPL, cerebellum posterior lobe; FG, fusiform gyrus; Hip, hippocampus; IFG, inferior frontal gyrus; SFG, superior frontal gyrus; Prc, precuneus.

Since the different fALFF areas might be utilized as markers to separate the RRMS from the HGs, the mean fALFF values of these different areas were extracted and used for ROC curve to explore whether the fALFF have the potential ability to distinguish the RRMS from the HGs. In the present study, the ROC analysis revealed that the seven areas exhibiting abnormal fALFF values showed two good and five fair AUC values (0.77 ± 0.03, 0.729~0.822) to discriminate the two groups (Figures 5A,B; Table 3). Further diagnostic analysis demonstrated that the mean beta values of these brain areas demonstrated low-high degree of sensitivities (74.93% ± 12.52, 57.7~92.6%) and specificities (74.84% ± 13.39, 59.3~96.3%). Next, we investigated the discriminatory power of the combinations with more brain regions (≥2) and found that combining with more brain regions could improve the discriminatory power. Each of the four combinations with more than five brain regions exhibiting abnormal fALFF received the same best discriminatory power to distinguish the two groups with a sensitivity of 96.3% and a specificity of 88.5% (Figure 5C; Table 3). These findings indicate that the fALFF parameter may serve as a potential marker to distinguish the RRMS from the HGs.

**Table 3:** ROC curve analysis for the fALFF difference in brain areas between RRMS and HGs.

**fALFF index**	**ROC curve**			
	**AUC**	**Sensitivity**	**Specificity**	**Cutoff value**
R_CPL	0.762	65.4%	88.9%	−0.3908
R_FG	0.744	84.6%	59.3%	−1.0845
L_Hip	0.822	92.6%	73.1%	−0.6546
L_IFG	0.801	57.7%	96.3%	0.4447
L_SFG	0.788	69.2%	74.1%	0.4163
R_Prc	0.746	70.4%	69.2%	1.3291
LR_SFG	0.729	84.6%	63%	0.3984
L_Hip + L_IFG + L_SFG + R_Prc + LR_SFG	0.944	96.3%	88.5%	0.4518325
R_CPL+ L_Hip + L_IFG + L_SFG + R_Prc + LR_SFG	0.943	96.3%	88.5%	0.4718962
R_FG+ L_Hip + L_IFG + L_SFG + R_Prc + LR_SFG	0.944	96.3%	88.5%	0.4590561
R_CPL+ R_FG+ L_Hip + L_IFG + L_SFG + R_Prc + LR_SFG	0.943	96.3%	88.5%	0.4718186

*ROC, receiver operating characteristic; AUC, area under curve; R, right; L, left; RRMS, relapsing-remitting multiple sclerosis; HGs, healthy groups; CPL, cerebellum posterior lobe; FG, fusiform gyrus; Hip, hippocampus; IFG, inferior frontal gyrus; SFG, superior frontal gyrus; Prc, precuneus*.

## Discussion

The study is the first to utilize the fALFF analysis to identify the RRMS-related abnormal regional brain activity and their correlations with demographic characteristics. RRMS was associated with widespread changes in brain areas, including cerebellum, orbitofrontal cortex, dorsolateral prefrontal cortex, supplementary motor area, and fusiform gyrus with higher fALFF values, and left hippocampus and precuneus in the occipital cortex with lower fALFF values. Recently, the ROC curve analysis has been widely applied into the exploration of the reliability and the potential of one neuroimaging approach to serve as an indicator in distinguishing one group from the other group (8, 16, 29). In the present study, the ROC curve revealed excellent AUC values for the combined areas, and further diagnostic analysis demonstrated that these combinations alone discriminated between the RRMS and the healthy subjects, with high degree of sensitivities and specificities. Thus, the resting-state fALFF analysis might serve as a potential biological indicator to characterize the abnormal regional brain activity in RRMS. Furthermore, in the RRMS group, the supplementary motor area negatively correlated with EDSS score, which may suggest that the motor cortex could predict the disability status of RRMS.

In the present study we found that the altered regional brain activity of the brain areas mainly manifested as increased fALFF values. There are two prevalent speculations (32). One explanation of this finding could be interpreted as brain compensation, in which the brain utilizes additional resources to help achieve the same level of performance as before. RRMS, disseminated in axonal degeneration and inflammatory demyelination in the central nervous system, is relative to the extensive white matter and gray matter damage (1, 2, 33). Another explanation of this finding could be interpreted as an enhanced neural effort to offset the noxious effects on the human brain function and structure.

RRMS is characterized by motor system disorder (1, 2), and extensive involvements of disease-related cortical changes may lead to several clinical symptoms, such as sensorimotor and cognitive deficits (2). The symptoms-related abnormal regional resting-state brain activity of RRMS may emerge ahead of the presence of visible brain lesions on T_2_WI (22). These regional areas covered the motor and visual systems. The cerebellum posterior lobe(s) works with cerebrum to complete several advanced cognitive and movement functions (34–36). The cerebellum posterior lobe is associated with regulation of coordinating movement (16, 23, 37). The cerebellar circuit has been associated with motor control function and motor behavior (32, 38). Abnormal regional resting-state brain activity, long-range functional connectivity, and gray matter atrophy in the motor systems (such as cerebellum) were also observed in the RRMS (39–41). The notion of dysfunctional neural integration in the cerebellum is supported by several positron emission tomography studies with decreased glucose metabolism in the bilateral cerebellum in early RRMS, which was ascribed to the remote effect of demyelinating lesions (42, 43). In support of these findings, our study showed altered regional brain activity in the cerebellum and supplementary motor area. Furthermore, the motor system correlated with the disability status of RRMS. Therefore, we speculated that the altered fALFF in the motor-related areas may be interpreted as functional deficit of the motor system caused by the RRMS, which may explain, at least partially, the motor system disorder of RRMS.

Cognitive function impairment may be involved with 43~70% of RRMS patients (44, 45). Previous studies primarily focused on how brain volume alteration (e.g., hippocampal) or T2 lesion load affect cognitive function (46, 47). These changes showed robust correlations with cognitive function (48, 49) and appear to be important predictors for cognitive function impairment in RRMS (50). In the present study, we explored how regional functional brain activity alterations of advanced cognitive function-related areas affect cognitive function. Here, we found fALFF differences in several cognitive-related areas (hippocampal, dorsolateral prefrontal lobe, and orbitofrontal cortex) in the RRMS relative to the HGs. The hippocampal and dorsolateral prefrontal areas are associated with advanced cognitive function, such as working memory (51–55). The orbitofrontal cortex (OFC), connected with prefrontal and deep structures known to mediate sensorimotor processing, motivation, and self-evaluation, is thought to be responsible for mediating the interactions between emotional processes and cognitive functions (56, 57) and play a significant role in fatigue, executive functions, attention, and motivation (58–60). Altered fALFF values of these advanced cognitive function-related areas may indicate cognition deficit of RRMS.

## Conclusions

In summary, the fALFF parameter may serve as an early potential biological marker to describe the abnormal regional brain activity associated with RRMS, ahead of visible brain lesion. Our study demonstrated that RRMS is associated with abnormal regional brain activity deficits in motor- and cognitive-related areas. These findings may provide intriguing insights into guide tailored treatment decisions and help provide us with a more comprehensive understanding of the pathophysiology of RRMS. However, the small number of subjects may be a major limitation; a larger number of sample sizes are necessary to corroborate our findings.

## Data Availability Statement

The raw data supporting the conclusions of this manuscript will be made available by the authors, without undue reservation, to any qualified researcher.

## Ethics Statement

All volunteers participated voluntarily and were explained the purposes, methods, and potential risks. Before the MRI session, all subjects voluntarily signed their informed consent form. This study was approved by the Human Research Ethics Committee of HwaMei Hospital, University of Chinese Academy of Sciences. No any additional considerations of the study in cases where vulnerable populations were involved (for example minors, persons with disabilities or endangered animal species).

## Author Contributions

X-FD and JL conceived and designed the whole experiment. Q-FH and Y-JW collected the data. JL took responsibility for the integrity of the data, the accuracy of the data analysis, and the statistical data analysis. X-FD wrote the main manuscript text. All authors contributed to the final version of the paper and have read, as well as, approved the final manuscript.

### Conflict of Interest

The authors declare that the research was conducted in the absence of any commercial or financial relationships that could be construed as a potential conflict of interest.
